# Outcomes of Retinal Detachment Surgery in Eyes with Chorioretinal Coloboma

**Published:** 2010-10

**Authors:** Alireza Ramezani, Mohammad-Hossein Dehghan, Ahmad Rostami, Hamid Ahmadieh, Morteza Entezari, Masoud Soheilian, Mohsen Azarmina, Siamak Moradian, Mehdi Yaseri

**Affiliations:** Ophthalmic Research Center, Shahid Beheshti University of Medical Sciences, Tehran, Iran

**Keywords:** Coloboma, Retinal Detachment, Vitrectomy, Scleral Buckle, Intraocular Pressure

## Abstract

**Purpose:**

To report the anatomical and functional outcomes of surgery for retinal detachment associated with chorioretinal colobomas.

**Methods:**

In this retrospective interventional case series, 28 eyes of 28 patients (including 18 male subjects) who had undergone surgery for retinal detachment associated with chorioretinal colobomas were evaluated regarding the type of intervention, final visual acuity and anatomical outcomes, as well as complications. Cases with less than 3 months of follow-up were excluded.

**Results:**

Primary surgery included vitrectomy in 25 (89.3%) and scleral buckling in 3 (10.7%) eyes. The internal tamponade used in eyes undergoing vitrectomy was silicone oil in 23 (92%) eyes and 20% sulfur hexafluoride (SF6) in 2 (8%) eyes. Silicone oil was removed in 11 eyes (45.8%). The mean number of operations per eye was 1.57±0.74, mean follow-up was 40±36 months, and the retina remained attached in 26 eyes (92.9%) at final follow-up. Mean preoperative visual acuity was 2.33±0.55 (range, 1.15–2.9) logMAR which significantly improved to 1.72±0.9 (range, 0.09–3.1) logMAR postoperatively (P < 0.001), however, final median visual acuity was counting fingers at 2 m. The most common complications were cataracts (100%) and ocular hypertension (46.4%).

**Conclusion:**

The most prevalent surgical procedure for treatment of retinal detachment associated with chorioretinal coloboma was pars plana vitrectomy and the most frequently used tamponade was silicone oil. Although anatomical success was satisfactory, functional outcomes were not encouraging which reflects the complexity of the condition and associated abnormalities.

## INTRODUCTION

Coloboma is a congenital defect of the eye caused by improper closure of the embryonal fissure. This condition occurs in 0.14% of the general ophthalmic population.^1^ Retinal detachment (RD) has been reported to occur in 8–50% of these patients.^2^ If RD is secondary to retinal breaks outside the colobomatous area, it can be treated by scleral buckling; however, when the coloboma is etiologically responsible for RD,^3^ management options are different.^4^

Outcomes of surgery for RD accompanying a chorioretinal coloboma are not as satisfying as that in the general population. Anatomical success rates of surgery in such eyes range from 40%^5^ to 100%^6^. Anatomical alterations in colobomatous eyes such as retinal thinning, staphylomatous and thinned sclera, and involvement of the disc and macula, as well as lack of choroid and retinal pigment epithelium make the operation technically difficult and hence the prognosis less satisfactory.^1^

In this study, we report the method of operation, as well as anatomical and functional outcomes in eyes undergoing surgery for colobomatous RD.

## METHODS

This retrospective study was based on existing data of patients with RD accompanying a chorioretinal coloboma who underwent surgery at two tertiary ophthalmic referral centers in Tehran, Labbafinejad Medical Center and Imam Hossein Medical Center, over a period of 10 years from 1997. The study was approved of by the Review Board/Ethics Committee of the Ophthalmic Research Center. Any patient with less than 3 months of follow-up was excluded.

Patient demographics, pre- and postoperative examinations, and the type of surgery, as well as postoperative complications were recorded in the data sheet. Snellen visual acuity (VA) was recorded and converted to logarithm of minimum angle of resolution (logMAR) for statistical analysis. Ophthalmic examinations included slitlamp and indirect ophthalmoscopic examinations. Intraocular pressure (IOP) was measured by Goldmann applanation tonometry. Anatomical success was defined as stable retinal reattachment posterior to the band or buckle.

Statistical analysis was performed using SPSS software (version 15; SPSS Inc., Chicago, USA). Qualitative variables were described using percentages; quantitative data were defined using means and standard deviations. The paired t-test was used for comparing pre- and postoperative values. In order to compare median VA in patients with different degrees of colobomatous involvement, the Kruskal-Wallis test was used. Level of statistical significance was set at 0.05.

## RESULTS

Data from 28 eyes of 28 patients who had undergone surgery for RD accompanying chorioretinal colobomas were evaluated. Involvement of the right and left eyes was equal. Mean patient age was 32.6±14.2 (range, 4 to 61) years and mean duration of symptoms prior to referral was 44.8±53.3 (range, 5 to 180) days. Patient demographics as well as baseline clinical characteristics are shown in table 1; specifications of the operations are presented in table 2.

Three patients had history of cataract surgery and intraocular lens implantation, of whom two underwent intraocular lens removal during vitrectomy. Two other patients had history of scleral buckling (SB).

Initial VA ranged between light perception and counting fingers at 3.5 m. The mean number of involved quadrants (extent of RD) was 2.96±0.79. All eyes without macular involvement by the coloboma had a detached macula at presentation. Retinal breaks were detected in 10 eyes (35.7%) preoperatively; 3 of these eyes underwent SB and 7 others (including giant tear, dialysis and multiple breaks, each in one eye) underwent vitrectomy. Preoperatively, proliferative vitreoretinopathy (PVR) grade C was present in 12 (40.8%) eyes. Ten eyes had posterior PVR only, involving 12, 9, 6, and 4 clock hours, respectively, in 1, 2, 4, and 3 eyes. The other two eyes had only anterior PVR in 6 and 3 clock hours.

Three patients (10.7%) underwent primary SB surgery (two with silicone tire No. 276 plus band No. 240, and one with a 180 degree circumferential sponge No. 507). One of these three patients experienced retinal re-detachment secondary to PVR and underwent lensectomy plus vitrectomy with C3F8, followed by repeat vitrectomy with silicone oil (SO) tamponade.

Twenty-five eyes (89.3%) underwent vitrectomy, of which, two had previously undergone SB surgery at other centers. Vitrectomies were performed in a standard fashion employing a three-port 20-gauge pars plana approach. Encircling band (No. 240) placement and pars plana lensectomy (including lens capsule removal) were performed in association with vitrectomy in 92% (23 eyes) and 90.9% (20 of 22 of phakic eyes), respectively. Intraocular lens removal was performed in two eyes. All eyes underwent intraoperative laser therapy around the colobomatous area and retinal breaks followed by air/fluid exchange. In three eyes, retinal breaks were detected during the operation. Intraocular tamponade included 20% sulphur hexafluoride (SF6) in 2 (8%) eyes and SO in 23 eyes (92%).

Retinal reattachment was achieved in all eyes at the end of the procedures and in the early post-operative period. Overall, the mean number of procedures (including SO removal) was 1.57±0.74 for each eye; 16 eyes (57.1%) underwent surgery once, 7 eyes (25.1%) were operated twice, and 5 eyes (17.8%) required surgery three times.

SO removal was performed in 11 of 24 eyes (45.8%). The reasons for retaining SO (13 eyes) were surgeons’ preference (11 eyes) and patients’ non-compliance for removal (2 eyes). SO removal resulted in retinal re-detachment in 3 eyes. All 3 eyes underwent repeat vitrectomy with SO tamponade which was anatomically successful. Retinal re-detachment occurred in two eyes with SO in place. Re-operation was not scheduled for them due to poor visual prognosis.

Mean preoperative VA was 2.33±0.55 (range, 1.15 to 2.9) logMAR which significantly improved to 1.72±0.9 (range, 0.09 to 3.1) logMAR postoperatively (P<0.001). Maximum and minimum preoperative VA was counting fingers at 3.5 m and light perception, respectively; these improved to 20/60 and no light perception after intervention. The change in median VA from hand motions to counting fingers at 2 m after the operations was also significant (P=0.001).

The distribution of Snellen visual acuity based on the site and extent of the coloboma is presented in table 3. Final median VA in eyes without colobomatous involvement of the disc and macula (counting fingers at 4 m) was better than eyes with disc colobomas (counting fingers at 2.5 m) and those with disc and macular colobomas (light perception and hand motions). The difference in final VA among these three categories was statistically significant (P=0.01).

Eventually and with a mean follow-up duration of 40±36 (range, 3 to 105) months, the retina remained attached in 26 eyes (92.9%) and detached in 2 eyes (7.1%). The retina remained attached in the two eyes for which SF6 was used for internal tamponade.

### Postoperative Complications

Two patients undergoing SB were phakic and the crystalline lens was preserved in two patients in the vitrectomy group. Cataract progression was observed in all 4 eyes necessitating cataract surgery in 2 eyes. The encircling band was removed in one eye because of band extrusion.

Of 3 eyes initially undergoing SB, IOP rise occurred in the only eye requiring vitrectomy plus C3F8 injection. In the vitrectomy group, 12 eyes (48%) developed high IOP necessitating the use of glaucoma medications. The tamponade used in these eyes was SF6 in 2 and SO in 10 eyes. Of the latter 10 eyes, five had retained SO but the other five had undergone SO removal 4 to 20 months after the procedure, when IOP rise occurred. IOP was controlled with medications in 8 eyes including the two eyes with SF6. However, the glaucoma was refractory to medical therapy in 4 eyes including 2 eyes with removed SO and 2 with retained SO. Of these 4 eyes, 2 with 20/120 and counting fingers at 3m vision underwent glaucoma surgery. The other 2 eyes were not expected to regain useful vision (one with light perception vision and one with recurrent RD) and were thus only observed.

## DISCUSSION

Mean follow-up in this study (40 months) was longer than that of some other large studies (13.4^5^ and 14^7^ months) reporting outcomes of retinal detachment surgery for colobomatous RD. The rate of final retinal reattachment in our study was 92.9% achieved with a mean number of 1.6 operations per eye which compares favorably with other reports at 81.2%^5^, 81.8%^4^, 87.5%^8^, and 100%^9^–^11^. These studies however, included only vitrectomized colobomatous eyes while excluding eyes undergoing SB. The favorable anatomical success rate in our study reveals that despite the presence of distinct abnormalities, outcomes of vitrectomy with SO tamponade in colobomatous eyes is as good as their non-colobomatous counterparts.

The present study demonstrated that despite a good anatomical success rate and significant visual improvement, final visual acuity was not satisfactory. Visual acuity equal to or better than 20/400 was achieved in only 35.7% of operated eyes in the current series, however, this level of vision was reported in 69.9%^5^ and 78.4%^7^ of cases in two large studies reporting on 85 and 42 cases. Such discrepancy may be due to a larger number of eyes with disc and macular colobomas or late referral. Colobomatous involvement of the disc and/or macula is a crucial prognostic factor in terms of functional outcomes. In the current study, final median VA was significantly better in eyes without disc and macular involvement. Final median VA in our study was counting fingers at 2 m which was less than that reported by Pal et al^7^ (20/200).

Although SB can be considered as a treatment modality in eyes with colobomatous RD,^7^ reported anatomical outcomes in the literature (35% to 57%) have not been satisfactory. This is due to difficulty in locating the retinal breaks, their posterior location, and inability to create adequate chorioretinal adhesions around the coloboma by cryo or laser. Scleral buckling has been performed using an encircling element or with two radial buckles around the colobomatous area as described by Wang and Hilton^12^ with a success rate of only 35%. Jesberg and Schepens^1^ believed that colobomatous eyes are not suitable for SB surgery. Most reports on SB surgery in colobomatous eyes date back to decades ago, e.g. 1961^1^, 1981^13^, 1985^12^. There has been a decline in the trend toward SB operations in colobomatous eyes; however, in case of a definite peripheral break, SB may still be considered as the first choice.^7^ Encircling SB was the preferred method in only 3 out of 28 eyes (10.7%) in our study.

The preferred surgical procedure in our study was vitrectomy in 89.3% of eyes. The possibility of finding breaks is higher with vitrectomy. Vitrectomy with SO tamponade was successfully employed for colobomatous RD for the first time in 1983.^14^ Since then, it has frequently been used by different experts.^4^,^8^–^10^ All eyes in our study, except those with previous SB surgery, received an encircling band at the time of vitrectomy. This procedure has been used in fewer cases in similar studies. In the study by Gopal et al^4^, 82.4% of vitrectomies included an encircling element. Without referring to any statistics, the authors concluded that placing an encircling band had no beneficial effect. In the report by Pal et al^7^, only 38% of eyes received an encircling element.

Lensectomy was performed in 90.9% of phakic eyes in our study. Lensectomy in addition to vitrectomy was performed in 71.4%^7^ and 83.5%^5^ of eyes with colobomatous RD in two other reports. To achieve good visualization during vitrectomy, removal of a cataractous lens is necessary. It may also help the surgeon trim the vitreous base and facilitate management of pathologies in this area. The only two eyes that remained phakic during vitrectomy developed cataracts later. This highlights the preference of lens removal during vitrectomy in colobomatous eyes. In this study, the surgeons did not intend to preserve the lens capsule. The rationale was to achieve higher success rates in such complicated eyes, of which many suffered from PVR as well. Additionally, because of poor visual potential, preservation of the lens capsule may have been unnecessary for simultaneous or future implantation of an intraocular lens.

Silicone oil has been preferred for tamponade in colobomatous RD repair by most surgeons.^4^,^5^,^8^–^10^ In the study by Gopal et al^5^, it was used in 80 of 85 eyes (94%); 3 of the 5 eyes who received gas at the end of the operation, experienced a retinal re-detachment. The authors concluded that gas is not a suitable tamponade because these eyes require a long effect over the entire edge of the coloboma. Nonetheless, the authors of another study recommend long-acting gas tamponade (C3F8) if the eye does not demonstrate severe proliferative changes.^6^ In our study, only two eyes were treated by SF6 and neither developed retinal re-detachment. However, it should be noted that neither our study nor others, had enough power to compare the use of SO versus gas during vitrectomy for colobomatous RD.

Silicone oil removal was performed in nearly half of our patients which is comparable to other studies.^5^,^7^ Retinal re-detachment occurred in 3 (27.3%) out of 11 eyes undergoing SO removal. In other reports, corresponding rates have been 10%^7^ and 15.6%^5^. Considering the 18.4% chance of retinal re-detachment after SO removal in non-colobomatous eyes^15^, it might be concluded that a coloboma per se is not an additional risk factor for retinal re-detachment following SO removal. The rate of retinal re-detachment in eyes with retained SO in our study was similar to another study reporting it at 11.9% due to progressive PVR.^7^

Most eyes underwent lensectomy during vitrectomy. Cataract progression occurred in all eyes which had remained phakic after the operation (vitrectomy or SB). Increased IOP was another common complication in our patients which occurred in 46.4% of vitrectomized eyes and was controlled with medications in most cases. In 14% (4 out of 13 cases), the glaucoma was refractory to medical therapy. The rate of IOP rise in the current study was more than other reports (16.3%^5^ and 11.9%^7^). This difference may be due to different timing for SO removal because SO emulsification has been considered as an important risk factor for IOP rise. SO removal has been recommended 6–8 weeks postoperatively in such cases.^5^

The shortcoming of this study is its retrospective nature. In addition, we were unable to compare vitrectomy with SB, and SO versus gas in the management of colobomatous RD.

This retrospective study adds more data to the literature regarding the preferred surgical options, as well as visual and anatomical outcomes of RD repair in eyes with chorioretinal coloboma. Based on this report and similar studies, vitrectomy with SO tamponade may be a good choice for management of colobomatous RD. Despite an acceptable anatomical result, the functional outcome is rather poor which reflects involvement of vital structures in the eye as well as amblyopia. Ocular hypertension is a complication that deserves careful pre- and postoperative evaluation.

## Figures and Tables

**Table 1:** Baseline clinical features of patients with colobomatous retinal detachment

Age (years)	32.6 ± 14.2 (range, 4–55)

Female/Male ratio	10/18

Eye (OD/OS)	14/14

Symptom duration (days)	46.8 ± 53.3 (range, 5–180)

Best-corrected visual acuity (logMAR)	2.33 ± 0.55 (range, 1.15–2.9)

Intraocular pressure (mmHg)	11.9 ± 5.2 (range, 4–30)

Cataract	
None	5 (17.9%)

Mild	18 (64%)

Moderate	2 (7.1%)

Severe	0 (0%)

Extent of retinal detachment	
1–2 quadrant	4 (14.3%)

2–3 quadrant	6 (21.4%)

3–4 quadrant	18 (64.3%)

Detected retinal breaks	10 (35.7%)

Previous interventions	
Surgery	6 (21.3%)

Laser	1 (3.6%)

Associated abnormalities	
Microphthalmia	13 (46.4%)

Iris coloboma	23 (82.1%)

Macular coloboma	9 (32.1%)

Disc coloboma	18 (64.3%)

Nystagmus	13 (46.4%)

OD, right eye; OS, left eye

**Table 2:** Operations performed for eyes with colobomatous retinal detachment

	N (%)	Mean ± SD
Number of operations per eye		1.57 ± 0.74
One	16 (57.1)	
Two	8 (28.6)	
Three	4 (14.3)	
Cataract surgery	2 (7.1)	
Scleral buckling	2 (7.1)	
Silicone oil removal	11 (39.3)	
Number of vitrectomy procedures		1.46 ± 0.79
One	16 (57.1)	
Two	8 (28.6)	
Three	4 (14.3)	
Barrier laser for the fellow eye	5 (17.9)	

N, frequency; SD, standard deviation

**Table 3:** Final Snellen visual acuity in 28 cases following colobomatous retinal detachment repair based on the extent of the coloboma

	Without disc or macular coloboma (n=10)	Only disc coloboma (n=9)	Both disc and macular coloboma (n=9)
	20/120	20/160	20/160
	LP	HM	NLP
	CF at 2 m	CF at 3 m	LP
	CF at 1.5 m	CF at 2 m	HM
	20/60	CF at 3 m	CF at 1.5 m
	CF at 4 m	CF at 2.5 m	HM
	20/60	CF at 1.5 m	LP
	20/200	CF at 1 m	NLP
	20/60	CF at 3.5 m	CF at 0.5 m
	CF at 4 m		

Median	CF at 4 m	CF at 2.5 m	HM

LP, light perception; CF, counting fingers; HM, hand motions; NLP, no light perception

## References

[1] Jesberg DO, Schepens CL (1961). Retinal detachment associated with coloboma of the choroid. Arch Ophthalmol.

[2] Steahly LP (1990). Retinochoroidal coloboma: varieties of clinical presentations. Ann Ophthalmol.

[3] Schubert HD (2005). Structural organization of choroidal colobomas of young and adult patients and mechanism of retinal detachment. Trans Am Ophthalmol Soc.

[4] Gopal L, Kini MM, Badrinath SS, Sharma T (1991). Management of retinal detachment with choroidal coloboma. Ophthalmology.

[5] Gopal L, Badrinath SS, Sharma T, Parikh SN, Shanmugam MS, Bhende PS (1998). Surgical management of retinal detachments related to coloboma of the choroid. Ophthalmology.

[6] Jalali S, Das T (1994). Selection of surgical technique for retinal detachment with coloboma of the choroid. Indian J Ophthalmol.

[7] Pal N, Azad RV, Sharma YR (2006). Long-term anatomical and visual outcome of vitreous surgery for retinal detachment with choroidal coloboma. Indian J Ophthalmol.

[8] Hanneken A, de Juan E, McCuen BW (1991). The management of retinal detachments associated with choroidal colobomas by vitreous surgery. Am J Ophthalmol.

[9] Corcostegui B, Guell JL, Garcia-Arumi J (1992). Surgical treatment of retinal detachment in the choroidal colobomas. Retina.

[10] McDonald HR, Lewis H, Brown G, Sipperley JO (1991). Vitreous surgery for retinal detachment associated with choroidal coloboma. Arch Ophthalmol.

[11] Teoh SC, Mayer EJ, Haynes RJ, Grey RH, Dick AD, Markham RH (2008). Vitreoretinal surgery for retinal detachment in retinochoroidal colobomata. Eur J Ophthalmol.

[12] Wang K, Hilton GF (1985). Retinal detachment associated with coloboma of the choroid. Trans Am Ophthalmol Soc.

[13] Patnaik B, Kalsi R (1981). Retinal detachment with coloboma of the choroid. Indian J Ophthalmol.

[14] Gonvers M (1983). Temporary use of silicone oil in the treatment of special cases of retinal detachment. Ophthalmologica.

[15] Lam RF, Cheung BT, Yuen CY, Wong D, Lam DS, Lai WW (2008). Retinal redetachment after silicone oil removal in proliferative vitreoretinopathy: a prognostic factor analysis. Am J Ophthalmol.

